# Patterns of Antibiotic Resistance in Urinary Tract Infections: A Retrospective Observational Study

**DOI:** 10.7759/cureus.62771

**Published:** 2024-06-20

**Authors:** Nitheesha Reddy Marepalli, Aneesh Rao Nadipelli, Rahul Jain Manohar Kumar Jain, Leela Sai Parnam, Anubhuti Vashyani

**Affiliations:** 1 Department of Internal Medicine, Dr. Patnam Mahender Reddy Institute of Medical Sciences, Hyderabad, IND; 2 Department of General Medicine, Government Siddhartha Medical College, Vijayawada, IND; 3 Department of General Medicine, Sri Manakula Vinayagar Medical College and Hospital, Puduchery, IND; 4 Department of General Medicine, Mediciti Institute of Medical Sciences, Hyderabad, IND; 5 Department of General Medicine, Indira Gandhi Medical College and Hospital, Shimla, IND

**Keywords:** empiric therapy, fluoroquinolones, nitrofurantoin, antibiotic resistance, urinary tract infection

## Abstract

Background: Urinary tract infections (UTIs) are among the most common bacterial infections, and antibiotic resistance complicates empiric treatment. This study aimed to describe recent resistance patterns among uropathogens in a tertiary-care teaching hospital to optimize empiric UTI management.

Methods: This retrospective observational study included 280 patients diagnosed with UTIs at the Dr. Patnam Mahender Reddy Institute of Medical Sciences, Hyderabad, over a six-month period from June 2023 to November 2023. Urine culture and antibiotic susceptibility data were collected from electronic medical records. Patient demographics, including age, sex, and comorbid diabetes, were recorded. Causative uropathogens and their resistance rates to commonly prescribed UTI antibiotics were analyzed. Empiric antibiotic treatment patterns and outcomes were talked about. These included clinical cure, recurrence, susceptibility match, and microbiologic eradication.

Results: The mean age of patients was 43.5 years, with 196 (70%) being female and 70 (25%) having diabetes. *Escherichia coli* caused 210 (75%) of UTIs, *Klebsiella pneumoniae* 42 (15%), *Proteus mirabilis* 14 (5%), *Enterococcus faecalis* 8 (3%), and *Staphylococcus saprophyticus* 6 (2%). *E. coli* resistance rates were 48% for ampicillin, 25% for ciprofloxacin, 18% for trimethoprim/sulfamethoxazole (TMP/SMX), and 5% for nitrofurantoin. *K. pneumoniae* resistance rates were 89% for ampicillin, 67% for ciprofloxacin, 44% for TMP/SMX, and 22% for nitrofurantoin. The most frequently prescribed antibiotic was nitrofurantoin (45%), then ciprofloxacin (35%). Clinical cure was achieved in 75% of cases. Recurrent UTIs within four weeks occurred in 25% of cases. Treatment matched urine culture susceptibility in 82% of patients.

Conclusion: The rising fluoroquinolone resistance highlights the need for current local data to guide empiric UTI treatment. Nitrofurantoin had low resistance rates and was an effective first-line therapy. Ongoing monitoring of resistance patterns in UTIs is essential to optimize antibiotic selection.

## Introduction

Urinary tract infections (UTIs) are one of the most prevalent bacterial infections worldwide, accounting for significant morbidity and high medical costs [1]. According to recent systematic reviews and meta-analyses, 150 million UTIs occur annually on a global scale. UTIs can involve the lower urinary tract, including acute uncomplicated cystitis and urethritis, or the upper urinary tract, including pyelonephritis, which carries the risk of sepsis [2,3]. The vast majority of community-acquired UTIs are caused by a limited range of Enterobacteriaceae, most commonly *Escherichia coli*, which accounts for 75%-95% of uncomplicated cystitis cases [4,5]. Other major uropathogens include *Klebsiella pneumoniae*, *Proteus mirabilis*, *Enterococcus faecalis*, and *Staphylococcus saprophyticus* [6]. The high prevalence of UTIs, along with escalating rates of antibiotic resistance among uropathogens, poses significant challenges for empiric antibiotic treatment [7].

The rising tide of antibiotic resistance worldwide threatens to undermine standard empiric antibiotic regimens for common infections, including UTIs. Surveillance studies providing updated local and regional resistance profiles are essential to inform clinical practice guidelines and optimize antibiotic selection [8,9]. However, resistance patterns can vary considerably depending on geographical region, patient population, healthcare settings, and even between outpatient versus inpatient cohorts. Ongoing monitoring of local resistance trends in UTIs is crucial given their high prevalence, potential for acute morbidity (including urosepsis and the risk of ascending kidney infection), and the expanding issues with multidrug-resistant Gram-negative pathogens such as carbapenem-resistant Enterobacteriaceae [10].

Fluoroquinolones such as ciprofloxacin have been the mainstay of empiric oral antibiotic therapy for uncomplicated cystitis in outpatients for many years, given their good bioavailability and historically broad-spectrum activity against most Gram-negative uropathogens [11]. However, there is a progressive increase in resistance to commonly used antibiotics worldwide. This includes resistance to fluoroquinolones, trimethoprim-sulfamethoxazole (TMP-SMX), cephalosporins, and even carbapenems. The escalating use of antibiotics is likely a primary factor exerting selection pressure, thereby driving antibiotic resistance development and spread [12]. Nitrofurantoin continues to exhibit relatively low resistance rates on a global scale and is widely recommended as a first-line oral antibiotic for the treatment of acute, uncomplicated cystitis by most major clinical guidelines [12]. Despite this, local resistance patterns can vary significantly due to differences in regional ecology, demographic factors, healthcare environments, and local antibiotic prescribing practices.

In urban clinic settings, where there is often a high prevalence of outpatient UTIs, it is crucial to have up-to-date surveillance data on the antibiotic susceptibility profiles of uropathogens isolated from urine cultures. This information is essential for guiding optimal empiric treatment choices and ensuring prudent clinical decision-making. Consequently, this study aimed to describe recent resistance patterns among common uropathogens isolated from patients diagnosed with UTIs in an outpatient setting at a tertiary-care teaching hospital. The specific objectives were to 1) identify the distribution of causative uropathogens, 2) determine the susceptibility profiles to key oral antibiotics used for empirical UTI treatment, and 3) assess clinical outcomes with different empiric antibiotic regimens selected based on updated local resistance data.

## Materials and methods

Study design and setting

This retrospective observational study was conducted at the Dr. Patnam Mahender Reddy (PMR) Institute of Medical Sciences, a 750-bed government medical college hospital located in Hyderabad, Telangana, India. The study spanned a six-month period, from June 2023 to November 2023.

Study population

The study population included all outpatients and inpatients diagnosed with UTIs based on urine culture results who attended the hospital during the study period.

Inclusion Criteria

Patients of all ages and genders are diagnosed with symptomatic UTIs based on clinical symptoms such as dysuria, increased frequency, and fever. Diagnosis is confirmed with a positive urine culture showing ≥10^5^ colony-forming units per milliliters of a single uropathogen.

Exclusion Criteria

Patients were excluded from the study if they had taken antibiotics within the previous two weeks, had indwelling urinary catheters or other forms of urinary tract instrumentation, exhibited polymicrobial growth in urine cultures, were diagnosed with asymptomatic bacteriuria, or were known or suspected to be pregnant.

Data collection

Data were retrospectively extracted from the microbiology records of urine culture and antibiotic susceptibility testing reports during the study period. Information was collected in a structured data collection form, including patient demographics (age, gender), the date of urine culture sample, department (outpatient/inpatient), causative uropathogen and colony count, antibiotic susceptibility testing results, empiric antibiotics prescribed as per clinical records, and treatment outcomes, such as clinical cure and persistence/recurrence based on follow-up visits.

Microbiological methods

Mid-stream urine samples were cultured on cysteine lactose electrolyte-deficient agar using a calibrated 0.001 mL loop. Bacterial identification was performed using conventional biochemical tests and automated systems like VITEK-2 (bioMérieux, Marcy-l'Étoile, France). Antibiotic susceptibility testing was conducted using the Kirby-Bauer disk diffusion method and interpreted as per Clinical and Laboratory Standards Institute 2022 guidelines. Quality control was ensured using standard American Type Culture Collection strains.

Statistical analysis

Data were analyzed using SPSS software version 25.0 (IBM Corp., Armonk, NY, USA). Descriptive statistics, including means and standard deviations for continuous variables and frequencies and percentages for categorical variables, were used to summarize the demographic and clinical characteristics of the study population. The antibiotic susceptibility profiles of the isolated uropathogens were assessed using the chi-square test for categorical variables to determine the statistical significance of resistance patterns. A p value of less than 0.05 was considered statistically significant.

Ethical approval

This study was approved by the Institutional Ethics Committee of the Dr. PMR Institute of Medical Sciences, Hyderabad, Telangana, India (approval number: PMR/GM/IEC/2023-8). All procedures performed in this study involving human participants were in accordance with the ethical standards of the institutional and/or national research committee and with the 1964 Helsinki Declaration and its later amendments or comparable ethical standards. The study was conducted with approval from the institutional ethics committee, and patient consent was waived due to the retrospective nature of the study.

## Results

Study population

The study encompassed 280 patients diagnosed with UTIs at the Dr. PMR Institute of Medical Sciences, Hyderabad, over a six-month period from June 2023 to November 2023. The demographic distribution revealed a predominance of female patients (70%), with males constituting the remaining 30%. The age spectrum ranged from 20 to 65 years, with a mean age of 43.5 years. A notable 25% of the patients had a documented history of diabetes mellitus (Table 1).

**Table 1:** Study population demographics

Characteristic	Value
Total patients	280
Female patients (%)	196 (70%)
Male patients (%)	84 (30%)
Age range (years)	20-65
Mean age (years)	43.5
Patients with diabetes (%)	70 (25%)

Causative uropathogens

Analysis of urine cultures identified the primary uropathogens responsible for UTIs. *E. coli* emerged as the predominant pathogen, isolated in 75% of cases, followed by *K. pneumoniae* (15%), *P. mirabilis* (5%), *E. faecalis* (3%), and *S. saprophyticus* (2%) (Table 2).

**Table 2:** Distribution of uropathogens

Uropathogen	Number of cases	Percentage (%)
E. coli	210	75%
K. pneumoniae	42	15%
P. mirabilis	14	5%
E. faecalis	8	3%
S. saprophyticus	6	2%

Antibiotic resistance rates

Antibiotic susceptibility testing, conducted via the Kirby-Bauer disk diffusion method, illuminated resistance rates among the identified uropathogens. For *E. coli* (n = 210), notable resistance was observed for ampicillin (48%), ciprofloxacin (25%), TMP/SMX (18%), nitrofurantoin (5%), ceftriaxone (parenteral) (8%), and piperacillin/tazobactam (parenteral) (3%) (Table 3). *K. pneumoniae* (n = 42) displayed even higher resistance rates, with ampicillin at 89%, ciprofloxacin at 67%, TMP/SMX at 44%, nitrofurantoin at 22%, ceftriaxone (parenteral) at 33%, and piperacillin/tazobactam (parenteral) at 11% (Table 4). Similar trends in resistance rates were evident for *P. mirabilis*, *E. faecalis*, and *S. saprophyticus*.

**Table 3:** Antibiotic resistance rates for E. coli (n = 210) TMP/SMX: trimethoprim/sulfamethoxazole

Antibiotic	Resistance (%)
Ampicillin	48%
Ciprofloxacin	25%
TMP/SMX	18%
Nitrofurantoin	5%
Ceftriaxone (parenteral)	8%
Piperacillin/tazobactam (parenteral)	3%

**Table 4:** Antibiotic resistance rates for K. pneumoniae (n = 42) TMP/SMX: trimethoprim/sulfamethoxazole

Antibiotic	Resistance (%)
Ampicillin	89%
Ciprofloxacin	67%
TMP/SMX	44%
Nitrofurantoin	22%
Ceftriaxone (parenteral)	33%
Piperacillin/tazobactam (parenteral)	11%

The resistance rates of uropathogens to various antibiotics were recorded, revealing significant challenges in empiric treatment. Ampicillin exhibited the highest resistance at 89%, followed by ciprofloxacin at 67% and TMP/SMX at 44%. Nitrofurantoin showed a comparatively lower resistance rate of 22%, making it a potentially effective option. Parenteral antibiotics, such as ceftriaxone and piperacillin/tazobactam, displayed resistance rates of 33% and 11%, respectively. These findings underscore the necessity for continuous monitoring and tailored antibiotic therapy to combat rising resistance levels.

The antibiotic resistance profiles of various uropathogens were evaluated, highlighting distinct resistance patterns. *P. mirabilis* showed resistance rates of 60% to ampicillin, 45% to ciprofloxacin, 30% to TMP/SMX, 15% to nitrofurantoin, 20% to ceftriaxone, and 10% to piperacillin/tazobactam. *E. faecalis* exhibited resistance rates of 55% to ampicillin, 40% to ciprofloxacin, 25% to TMP/SMX, 10% to nitrofurantoin, 15% to ceftriaxone, and 5% to piperacillin/tazobactam. *S. saprophyticus* presented with resistance rates of 50% to ampicillin, 35% to ciprofloxacin, 20% to TMP/SMX, 5% to nitrofurantoin, 10% to ceftriaxone, and 3% to piperacillin/tazobactam. These findings underscore the need for targeted antibiotic selection based on specific resistance patterns to effectively manage UTIs (Table 5).

**Table 5:** Antibiotic resistance rates for other uropathogens TMP/SMX: trimethoprim/sulfamethoxazole

Uropathogen	Ampicillin (%)	Ciprofloxacin (%)	TMP/SMX (%)	Nitrofurantoin (%)	Ceftriaxone (parenteral) (%)	Piperacillin/tazobactam (parenteral) (%)
P. mirabilis	60%	45%	30%	15%	20%	10%
E. faecalis	55%	40%	25%	10%	15%	5%
S. saprophyticus	50%	35%	20%	5%	10%	3%

Treatment patterns

The primary treatment for UTIs predominantly involves the administration of various antibiotics. Nitrofurantoin was the most frequently prescribed, accounting for 45% of cases, followed by ciprofloxacin (35%), TMP/SMX (15%), fosfomycin (5%), ceftriaxone (parenteral) (8%), and piperacillin/tazobactam (parenteral) (2%) (Table 6).

**Table 6:** Treatment patterns TMP/SMX: trimethoprim/sulfamethoxazole

Antibiotic	Percentage (%)
Nitrofurantoin	45%
Ciprofloxacin	35%
TMP/SMX	15%
Fosfomycin	5%
Ceftriaxone (parenteral)	8%
Piperacillin/tazobactam (parenteral)	2%

Outcomes

Analysis of treatment outcomes revealed a clinical cure rate of 75%, signifying successful resolution of UTI symptoms. However, at the four-week follow-up, 25% of patients experienced either persistent or recurrent UTIs. Notably, treatment aligned with urine culture susceptibility results in 82% of cases, highlighting the importance of tailoring treatment strategies based on individual resistance profiles. Furthermore, microbiologic eradication, assessed through a one-week follow-up urine culture, was achieved in 68% of patients (Table 7).

**Table 7:** Treatment outcomes UTIs: urinary tract infections

Outcome	Percentage (%)
Clinical cure rate	75%
Persistent/recurrent UTIs	25%
Treatment aligned with urine culture results	82%
Microbiologic eradication	68%

## Discussion

UTIs continue to be a prevalent public health concern, and understanding the dynamics of causative uropathogens, their antibiotic resistance profiles, and treatment outcomes is essential for informed clinical decision-making. The findings of this six-month retrospective observational study provide valuable insights into the multifaceted nature of UTIs and offer implications for empirical treatment strategies.

In line with global epidemiological trends, our study shows that *E. coli* is the most common uropathogen, with a prevalence of 75%. This is similar to what Akram et al. [13] and Bader et al. [14] found. Other important uropathogens like *K. pneumoniae*, *P. mirabilis*, *E. faecalis*, and *S. saprophyticus* are also included. This increases the variety of microbes that cause UTIs in our urban Indian setting [15]. This underscores the necessity for a nuanced and comprehensive approach to empirical therapy that acknowledges the spectrum of pathogens involved.

Our study exposes concerning antibiotic resistance rates within *E. coli* isolates. Notably, nearly half of the isolates exhibited resistance to ampicillin, and there was significant resistance observed against ciprofloxacin and TMP-SMX. These results are consistent with the rising trend of fluoroquinolone and TMP-SMX resistance noted in India by Akram et al. [13] and Bobbadi et al. [16]. The reported resistance rates in our study surpassed those documented in studies from Bangladesh and Brazil, underscoring the urgent need for tailored antibiotic stewardship strategies in our specific context [17,18].

*K. pneumoniae* exhibited notably high resistance levels, particularly against ampicillin and ciprofloxacin. These findings echo observations in North India by Goel et al. [19], indicating a broader regional concern of antibiotic resistance. Several studies [20,21] have shown that carbapenem-resistant *K. pneumoniae* is common in India. This shows how important it is to have antibiotic policies and keep an eye on things all the time to stop resistance from spreading.

Nitrofurantoin, recommended as the first-line oral treatment for simple cystitis by the World Health Organization (WHO), continued to demonstrate effectiveness against most uropathogens in our study [14]. According to Bader et al., for healthy nonpregnant women, the preferred antibiotics are nitrofurantoin for a five-day course, fosfomycin as a single dose, or pivmecillinam for five days. TMP-SMX and ciprofloxacin are not recommended due to their limited effectiveness, especially if they are recently used or for individuals at risk of certain infections. Alternative antibiotics include cephalexin, cefixime, fluoroquinolones, and amoxicillin-clavulanate. Options for resistant bacteria include cefepime, piperacillin-tazobactam, and carbapenems. In severe cases, intravenous antibiotics like piperacillin-tazobactam, carbapenems, and ceftazidime-avibactam may be necessary [14]. It is crucial to use antibiotics judiciously to prevent further resistance. However, the varying susceptibility profiles across different organisms underscore the importance of tailoring therapy based on individual culture results [22]. This emphasis on tailored therapy becomes particularly crucial amid rising antibiotic resistance rates, highlighting the need for precision in treatment approaches.

Limitations

Several limitations must be considered when interpreting these findings. First, the retrospective nature of the study could introduce inherent biases, affecting the accuracy and reliability of the data. Second, the relatively small sample size may restrict the generalizability of the results to broader populations. Furthermore, the absence of molecular characterization of resistance mechanisms limits the ability to gain a deeper understanding of the evolving landscape of antibiotic resistance. These factors underscore the need for cautious interpretation and suggest that further, more comprehensive research is necessary to validate these findings.

## Conclusions

This study enhances our understanding of UTIs by examining the prevalence of uropathogens, their resistance patterns, and treatment outcomes in a tertiary-care teaching hospital. The identified resistance trends emphasize the importance of continuous surveillance and awareness of local resistance patterns to guide empirical treatment decisions. Future strategies should integrate clinical, microbiological, and molecular data to refine treatment approaches and address the escalating challenge of antibiotic resistance in UTIs. Additionally, future research should explore innovative interventions to optimize empirical treatment and mitigate the impact of antibiotic resistance on UTI management in diverse clinical settings. The study findings support nitrofurantoin as highly effective against most uropathogens, aligning with WHO recommendations for simple cystitis treatment. Nitrofurantoin, fosfomycin, and pivmecillinam are endorsed as first-line options for healthy nonpregnant women, highlighting the limited efficacy of TMP-SMX and ciprofloxacin, especially in cases of recent use or specific infection risks. The study also discusses alternative antibiotics and specific options for resistant bacteria, emphasizing the need for prudent antibiotic use and tailored therapy based on individual culture results due to varying susceptibility profiles across different organisms.
